# Clinical characteristics and prognostic factors of hepatoblastoma in 316 children aged under 3 years – a 14-year retrospective single-center study

**DOI:** 10.1186/s12887-021-02630-2

**Published:** 2021-04-13

**Authors:** Tian Zhi, Wei-Ling Zhang, Yi Zhang, Hui-Min Hu, Dong-Sheng Huang

**Affiliations:** Department of Pediatrics, Beijing Tongren Hospital Capital Medical University, No. 1 Dongjiaominxiang Dongcheng District, Beijing, 100730 China

**Keywords:** Hepatoblastoma, Children, AFP, PRETEXT staging, Risk factor, Prognosis

## Abstract

**Background:**

The aim of the present study was to summarize the clinical characteristics of in children aged under 3 years and analyze the related factors affecting the prognosis.

**Methods:**

The clinical data of 316 children aged under 3 years (192 males and 124 females) who were admitted to Beijing Tongren Hospital with a pathological diagnosis of HB between May 2005 and May 2019 were analyzed retrospectively. The factors influencing the therapeutic effects on and survival of HB in children with HB were analyzed.

**Results:**

(1) The median age of the enrolled patients was 1.45 years. The most common initial symptom was an abdominal mass (69.0%). The average serum alpha-fetoprotein (AFP) level and platelet count at the initial visit were (97,406.5 ± 5022.8) ng/mL and (418 ± 206) × 10^9^/L, respectively. The epithelial type was the main pathological type (51.9%). According to the PRETEXT preoperative typing system, the most common stage was stage III (57.0%), whereas according to the postoperative Evans staging system, the most common stage was stage IV (41.8%). At the initial visit, 62 cases (19.6%) had vascular invasion, 52 cases (16.5%) had extrahepatic tumor extension, and 20 cases (6.3%) had tumor rupture. Distant metastasis occurred in 132 cases, and the most common metastatic site was the lung (80.3%). The incidence in East China was relatively high (35.4%). (2) The children were followed up until May 2020 (the median follow-up duration was 62 months). It was found that 194 patients had complete remission and 62 had partial remission. The Kaplan–Meier survival analysis showed that the overall survival was 95.3, 88.2, and 79.8% at 1 year, 3 years, and 5 years, respectively, and the event-free survival was 91.1, 83.2, and 75.1%, respectively. The Cox regression analysis showed that AFP level, platelet count, PRETEXT IV, vascular invasion, and distant metastasis at the initial visit were independent risk factors for the prognosis of children with HB (*p* < 0.05 in all).

**Conclusion:**

The prognosis of HB was correlated with the AFP level, platelet count, PRETEXT staging, vascular invasion, and distant metastasis at initial diagnosis.

## Background

As a rare cancer with an incidence of only 1.2–1.5/1000,000 in children, hepatoblastoma (HB) is usually diagnosed in children aged under 3 years [1]. However, HB is the most common malignant tumor in hepatic cancer in children, accounting for 60% of the primary hepatic cancer in children [2, 3]. With the continuous improvement in modern medical technology, the disease-free survival rate in low-risk children with HB can reach 80–90% [4]; however, the prognosis in high-risk pediatric patients, especially those with distant metastasis, remains poor. Therefore, it is vital to understand the risk factors affecting the prognosis of children with HB. In the present study, the clinical data of 316 children aged under 3 years who were diagnosed with HB by pathology between May 2005 and May 2019 in our single center were analyzed, and the curative effect of multidisciplinary combined therapy and the risk factors affecting the prognosis were investigated, with the aim of providing the corresponding intensive treatment for children with HB with different risk stratification and improving the prognosis.

## Methods

### Study subjects

The clinical data of 316 pediatric patients aged under 3 years who were admitted to Beijing Tongren Hospital affiliated to Capital Medical University between May 2005 and May 2019 with a diagnosis of HB by primary liver tumor pathology were collected and statistically analyzed. All cases had complete follow-up data, and all relevant examinations and treatments were approved by the guardians, who signed forms giving informed consent. The present study was approved by the Ethics Committee of Beijing Tongren Hospital affiliated to Capital Medical University.

#### Criteria for the histopathology and staging

According to the tumor tissue morphology analyzed by CHIC classification [5], two main histological types of HB occurred: epithelial and mixed epithelial/mesenchymal. The epithelial type was further divided into subtypes, such as fetal, embryonal, combined fetal and embryonal or small-cell undifferentiated type. The mixed type is characterized by the presence of some extra-mesenchymal elements, such as cartilage or osteoid. The PRETEXT staging system [6] proposed by the was adopted, and according to the anatomical structure of the liver and the number of liver segments involved, the patients with HB were divided into stages I to IV. After the operation, following the Evans staging system of Children’s Oncology Group (COG) [7], the patients were divided into stages I to IV according to the scope of tumor resection (Table 1).

**Table 1:** Different HB staging criteria for children with the disease

Stage	Phasing criteria
The PRETEXT preoperative typing system	
Stage I	Tumor confined to 1 liver segment, no tumor invasion in 3 adjacent liver segments
Stage II	The tumor involved 2 liver segments and the other 2 adjacent liver segments were not invaded by the tumor.
Stage III	The tumor involves 2 liver segments and the other 2 non-contiguous liver segments are not involved; or the tumor involves 3 liver segments.
Stage IV	The tumor involved 4 liver segments.
Annotation factors	P (portal invasion)
	V (hepatic vein/vessel invasion)
	F (multifocality)
	E (extrahepatic tumor extension)
	R (tumor rupture)
	M (distant metastasis)
The postoperative COG Evans staging system	
Stage I a	Complete resection of the tumor, histopathologic type of simple fetal type
Stage I b	Complete resection of tumor, histopathological types other than simple fetal type
Stage II	Tumor largely resected, with microscopic remnants
Stage III	Tumor with visual remnants, or basic resection with positive lymph nodes, or tumor rupture or peritoneal hemorrhage
Stage IV	Occurrence of distant metastases at diagnosis, regardless of complete resection of the primary lesion

#### The comprehensive therapeutic protocols

For children with suspected HB, the organ function and the size of mass were evaluated by the surgical department to ensure the resectability. If it was indicated that the patient was in PRETEXT I or partial stage II, the tumor should be resected first, and chemotherapy should be applied after the operation. For some pediatric patients with PRETEXT II, III, or IV, neoadjuvant chemotherapy was applied first, usually with 2–4 cycles of chemotherapy before the operation and 4–6 cycles of consolidation chemotherapy after the operation. The conventional first-line chemotherapy protocol comprised cisplatin + fluorouracil + vincristine (C5V protocol), cisplatin + adriamycin (PLADO protocol), or ifosfamide + carboplatin + pirarubicin + etoposide. For the cases with poor therapeutic effects of the first-line chemotherapy, or repeated recurrence and metastasis, individualized chemotherapy might be used, such as the protocol comprising irinotecan + cyclophosphamide + cisplatin + vincristine, etoposide + cisplatin + pirarubicin, or cyclophosphamide + cisplatin + pirarubicin, and the chemotherapy should be extended to 12–18 cycles (According to AFP changes, imaging changes and in vitro susceptibility test results, a personalized chemotherapy regimen was used). Mesna was utilized as a rescue after the administration of cyclophosphamide. Liver transplantation might also be considered for some pediatric patients with PRETEXT IV, portal vein invasion, or if the tumor is unresectable by the traditional surgery, and with postoperative tumor residual/recurrence. For patients with refractory HB, high-dose chemotherapy (melphalan + etoposide + cyclophosphamide) combined with might be used to prolong the survival. Moreover, targeted therapy, molecular biological therapy, and other individualized therapeutic methods might be applied.

#### Monitoring indicators, follow-up and evaluation criteria

The serum alpha-fetoprotein (AFP) level (normal range: 0 ~ 20 ng/mL) was detected before each cycle of chemotherapy. The peripheral blood routine test was conducted during chemotherapy. Imaging examinations (B-ultrasonography, CT) of the primary and/or metastatic lesions were performed after every two cycles of chemotherapy. The follow-up period was up to May 2020, and the follow-up was completed by returning to the hospital for re-examination and telephone follow-up. According to the results of follow-up, the clinical data, overall survival (OS) and event free survival (EFS) of the children were analyzed, and the potential risk factors affecting the prognosis were analyzed.

#### Criteria for the judgment of the therapeutic effect

Complete remission (CR): The tumor had disappeared completely after the treatment and there was no evidence of residual tumor in the imaging, together with the serum AFP being normal for more than 4 weeks. Partial remission (PR): The tumor had shrunk by more than 50%, without new focus, and the serum AFP had decreased significantly. Progressed disease (PD): The tumor volume had increased by more than 25%, with new tumor focus, or the AFP had increased or exceeded the normal value for two consecutive weeks during the treatment. Recurrence: After complete remission, it was confirmed by pathological biopsy that the tumor had appeared again, or there was clear imaging evidence and the serum AFP had increased three times within 4 weeks, or death [8].

#### Statistical analysis

SPSS 19.0 software was used for the data analysis. The measurement data were expressed as mean ± standard deviation, and the χ^2^ test was utilized for comparison between groups. In addition, the Kaplan–Meier method was utilized for the survival analysis. The log-rank test was employed for the comparison of survival rates among subgroups. The Cox regression model was utilized for multivariate risk analysis. *P* < 0.05 was considered statistically significant.

## Results

### Clinical characteristics

#### Clinical symptoms

The clinical data of the 316 pediatric patients enrolled in the present study are presented in Table 2. There were 192 males and 124 females, and the male/female ratio was 1.5. The age range was 0.08–2.92 years, with a median age of 1.45 years. In the present study, five cases were examined during the pregnancy (32–38 weeks) utilizing fetal B-ultrasonography, ed. that there was a hyperechoic mass in the liver area. The finding of a hepatic mass was diagnosed as HB by pathology after birth. The pathological types of 3 patients were epithelial type and 2 patients were mixed type. All five child patients were conceived naturally. Among the five cases, one case was born with very low birth weight, and two cases were born with low birth weight. In these cases, the birth weight was 1360 g, 1950 g, and 2130 g, respectively. In addition, among the five mathers, two mothers were pregnant with advanced age and had gestational hypertension, and one mother had a history of smoking. The onset of HB was insidious, and the most common sign was the abdominal mass, which was found in 218 cases (69.0%). The symptoms at the initial visit were as follows: abdominal pain and distention in 42 cases (13.3%); vomiting, poor appetite, and diarrhea in 25 cases (7.9%); fever and cough in 18 cases (5.7%); jaundice in 7 cases (2.2%); and emaciation and anemia in 6 cases (1.9%).

**Table 2:** Clinical characteristics of 316 children under 3 years of age with HB

	n	Ratio (%)
Gender		
Male	192	60.8
Female	124	39.2
Age (years-old)		
< 1	128	40.5
1–3	188	59.5
AFP at first consultation (ng/mL)		
≤ 100	17	5.4
101–1000	100	31.6
1001-1000,000	189	59.8
> 1000,000	10	3.2
Platelet count at first consultation (×10^9^/L)		
≤ 400	142	44.9
> 400	174	55.1
Pathological typing		
Epithelial type	164	51.9
Fetal type	61	37.2
Embryonal type	90	54.9
combined fetal and embryonal type	9	5.5
Small cell undifferentiated type	4	2.4
Mixed type	152	48.1
The PRETEXT staging system		
Stage I	9	2.8
Stage II	84	26.6
Stage III	180	57.0
Stage IV	43	13.6
The COG Evans staging system		
Stage I	9	2.8
Stage II	80	25.3
Stage III	95	30.1
Stage IV	132	41.8
Portal vein (P) invasion, Hepatic vein/vessel (V) invasion		
Y	62	19.6
N	254	80.4
extrahepatic tumor extension (E)		
Y	52	16.5
N	264	83.5
Tumor rupture (R)		
Y	20	6.3
N	296	93.7
Distant metastasis (M)		
Y	132	41.8
N	184	58.2
Multifocality (F)		
Y	74	23.4
N	242	76.6
Complete removal of the primary tumor		
Y	213	67.4
N	103	32.6

#### Laboratory indicators

The average AFP value at the initial visit was 97,406.5 ± 5022.8 ng/mL, with a maximum value of > 484,000 ng/mL and a minimum value of 43.6 ng/mL, which were all higher than the normal range. In 199 cases (63.0%), the AFP value was higher than 1000 ng/mL. The average platelet count at the initial visit was (418 ± 206) × 10^9^/L, with a maximum count of 1550 × 10^9^/L and a minimum count of 65 × 10^9^/L. Among these cases, 174 cases (55.1%) had a platelet count > 400 × 10^9^/L.

#### Histopathology and clinical staging

Regarding the pathological types, 51.9% (164/316) were the epithelial type and 48.1% (152/316) were the mixed type. Among the epithelial types, the majority of the cases were the embryonal type (90 cases, 54.9%), followed by the fetal type (61 cases, 37.2%), then the combined fetal and embryonal type (9 cases, 5.5%), and finally the small-cell undifferentiated type (4 cases, 2.4%). According to the PRETEXT preoperative staging system, the majority of cases in the present study were in stage III, accounting for 57.0%, whereas according to the postoperative COG Evans staging system, the majority were in stage IV, accounting for 41.8%.

#### Annotation factors

**Fig. 1:**
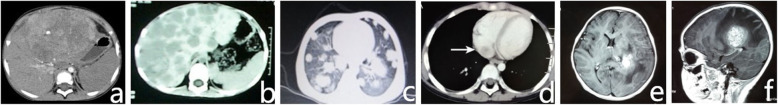
Imaging features of primary focus and different metastatic lesions of HB. **a** The abdominal CT in a patient with HB showed a large mass of the liver with visible local calcification. **b** The abdominal CT showed multiple intrahepatic metastatic foci. **c** Chest CT showed HB with multiple metastases of bilateral lungs. **d** HB with the right atrial metastasis thrombus. **e-f** The brain MRI showed HB with intracranial metastasis

**Fig. 2:**
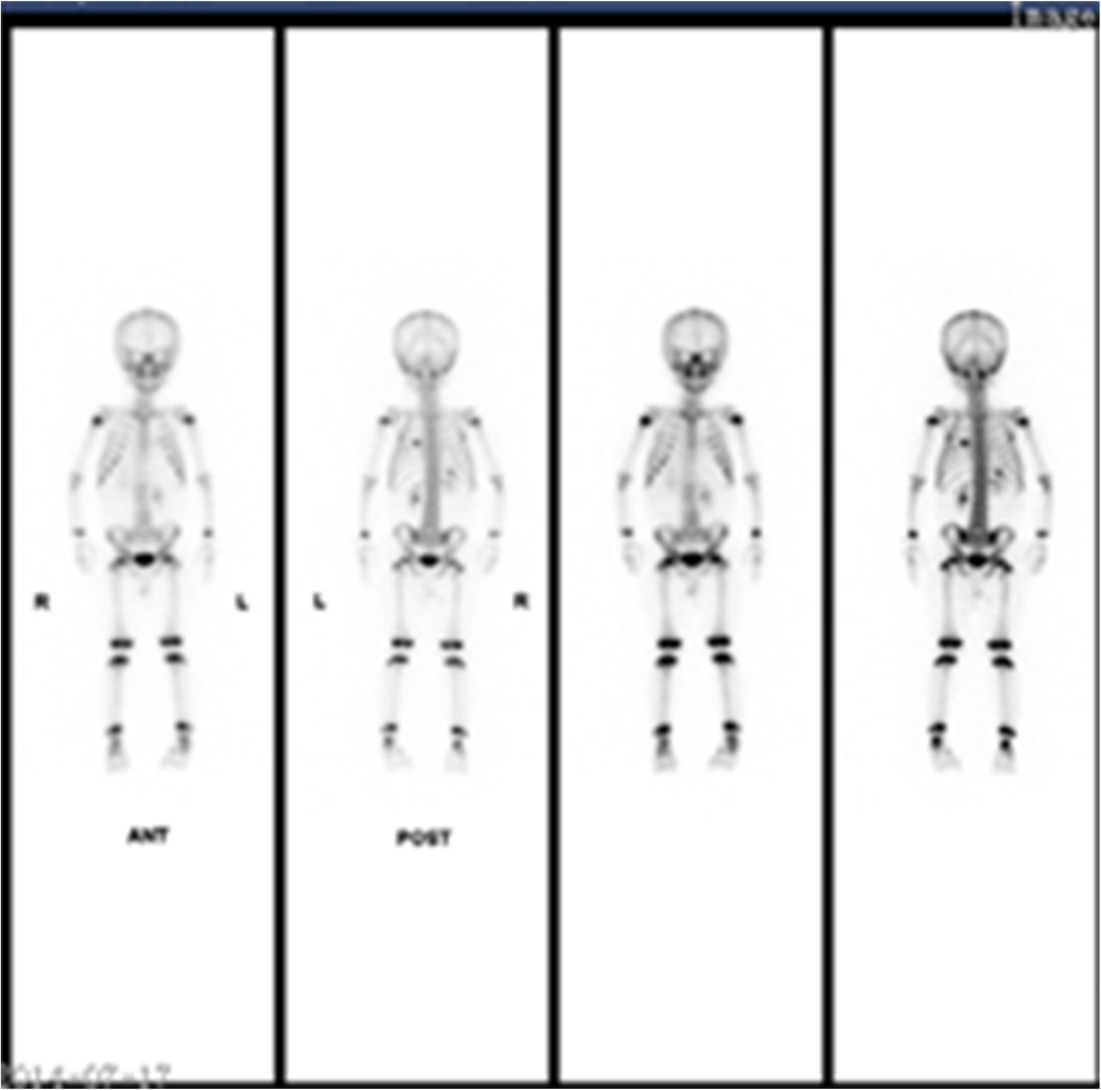
The whole body bone scan showed HB with bone metastasis, with a round radioactive distribution concentrated area in the left 7th rib and a patchy radioactive distribution increased area in the right 11th rib

**Fig. 3:**
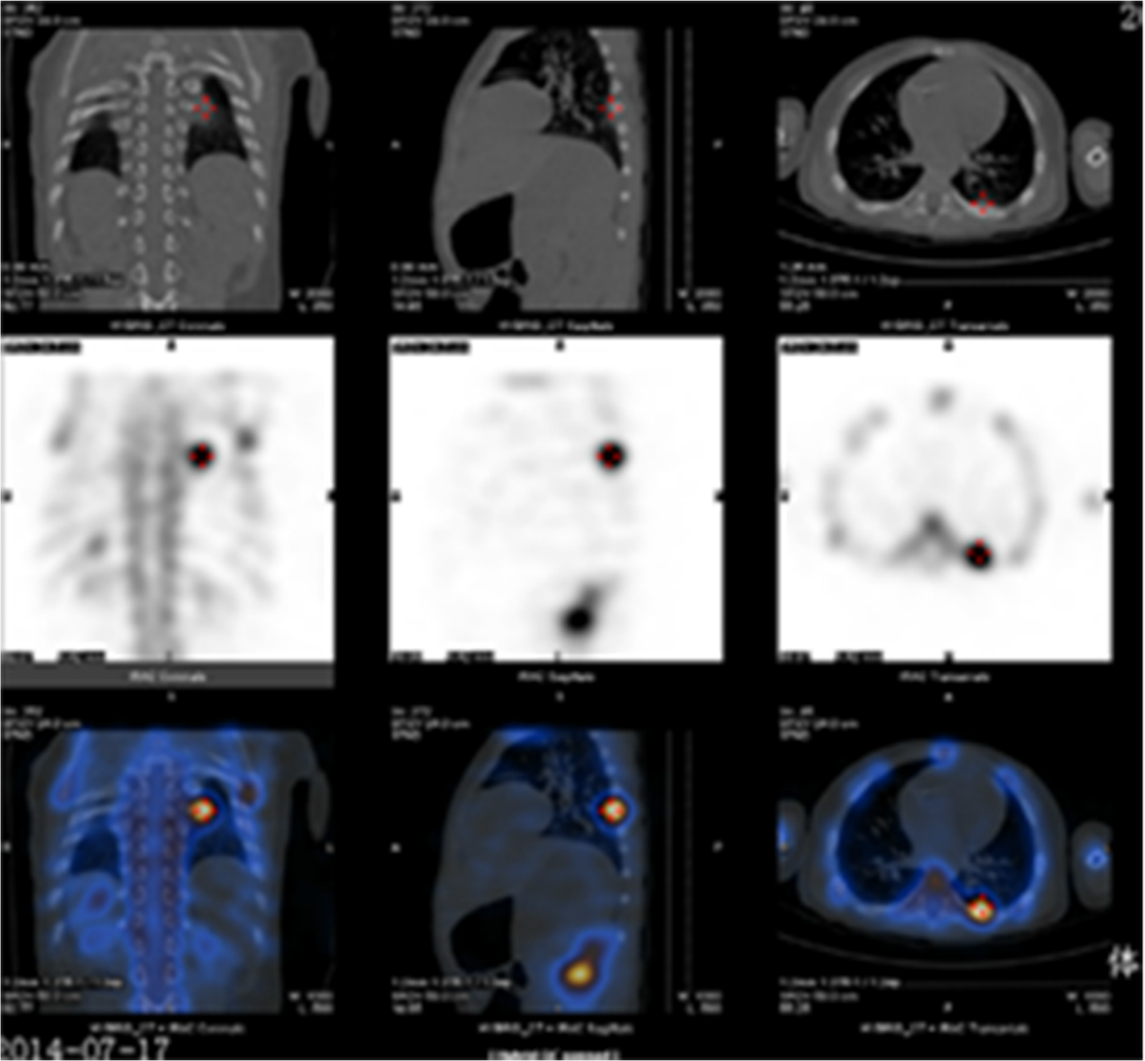
The whole body bone scan showed HB with bone metastasis, with a round radioactive distribution concentrated area in the left 7th rib and a patchy radioactive distribution increased area in the right 11th rib

At the initial visit, 62 cases (19.6%) had invasion to the portal vein, hepatic vein and vena cava (P + V), 52 cases (16.5%) had extrahepatic tumor extension (E), and 20 cases (6.3%) had tumor rupture (R). Among the 132 cases (41.8%) with distant metastasis (M) in the present study, the most common metastasis site was the lung, accounting for 80.3% (106/132), including 74 cases with unilateral lung metastasis (27 cases with left lung metastasis, 47 cases with right lung metastasis). In addition, there were 32 cases with bilateral lung metastasis, 84 cases with single marginal lung metastasis (79.2%), and 22 cases with marginal area combined with central region lung metastasis (20.8%). The other cases with metastasis were as follows: eight cases with intracranial metastasis, six cases with bone metastasis, four cases with diaphragm metastasis, four cases with right atrial thrombus, four cases with pleural metastasis, two cases with bone marrow metastasis, one case with intestinal and mesenteric metastasis, two cases with renal and adrenal metastasis, and two cases with intraspinal metastasis. Furthermore, 100 cases (75.8%) had metastasis in a single site, while 32 cases had remote metastasis in multiple sites (24.2%). Seventy-four cases (23.4%) were multifocality (F) (Figs. 1, 2 and 3).

#### Distribution of patients in different regions

**Fig. 4:**
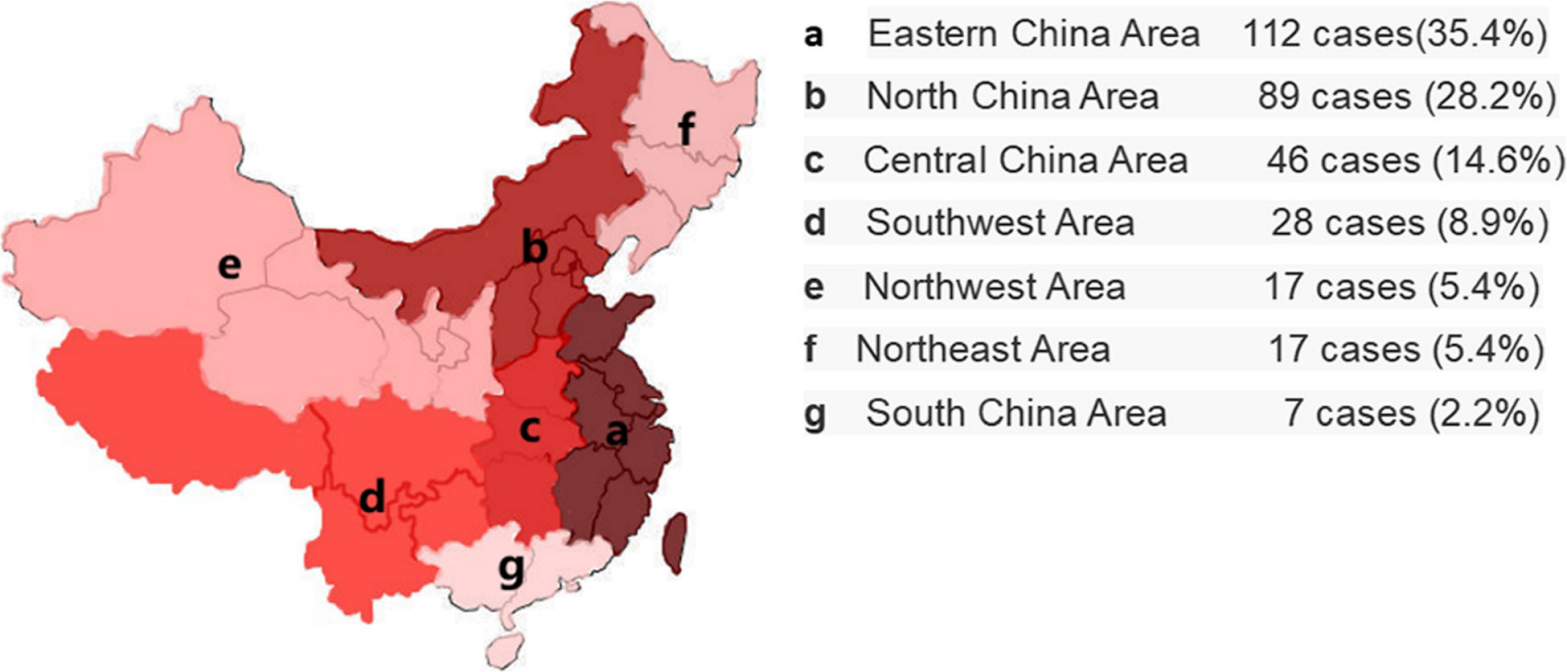
The incidence of HB in different regions of China by the present single-center statistics

The sources of patient referrals were widely distributed in this study. Eastern China accounted for the most cases, with 112 patients (35.4%), among which the three most common proviences of origin were Zhejiang (32 cases), Jiangsu (29 cases) and Shandong (24 cases). North China had the second highest number of patients, with 89 cases (28.2%), while the number of patients in the inland areas such as Northeast China and Northwest China was relatively low (Fig. 4).

### Follow-up and survival

**Fig. 5:**
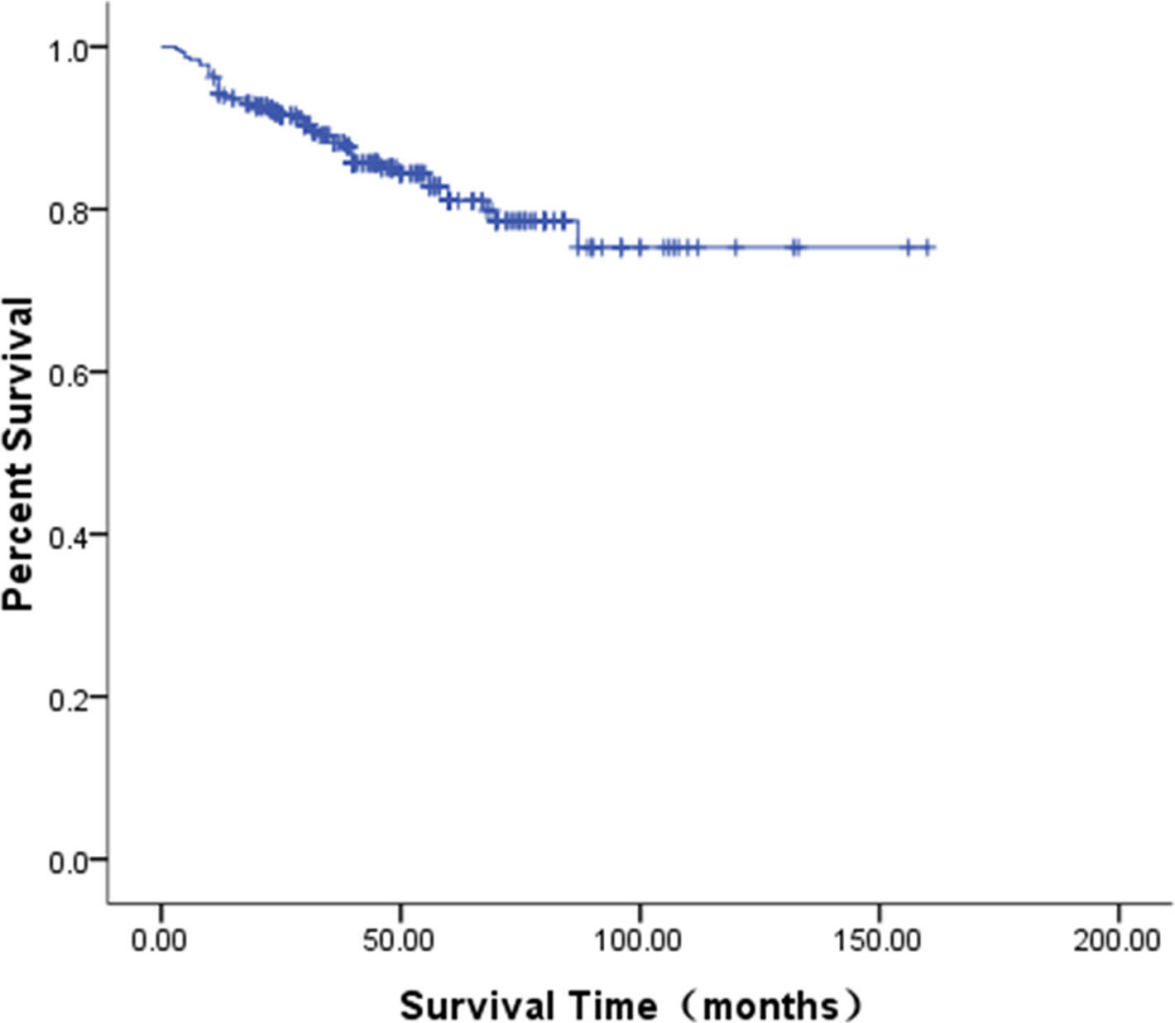
The OS curve of the 316 pediatric patients with HB

**Fig. 6:**
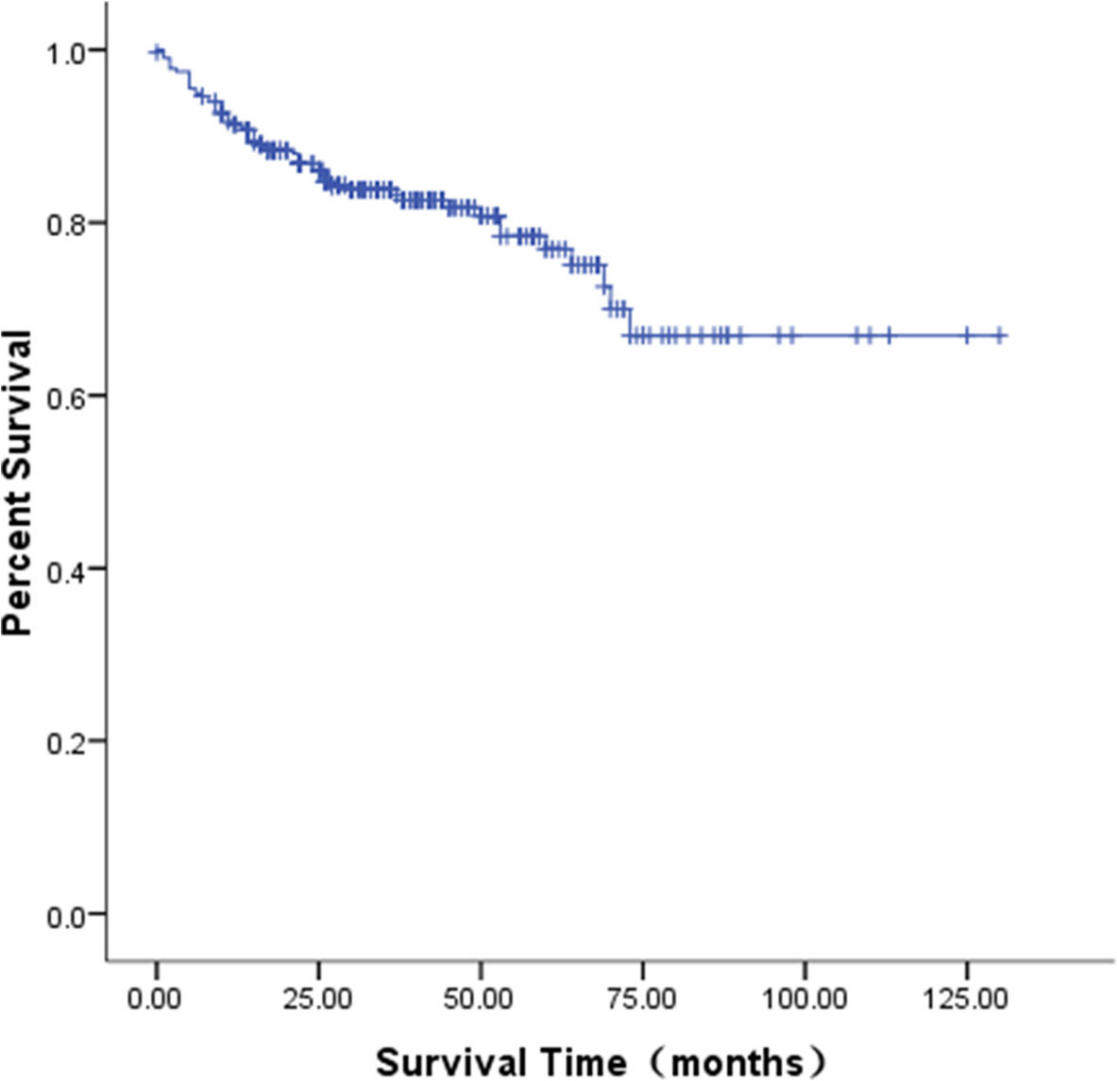
The EFS curve of the 316 pediatric patients with HB

The follow-up period was up to May 2020, with a duration of 1–165 months and a median duration of 62 months. In total, 194 patients achieved CR (among these cases, the pulmonary metastatic lesions in 2 patients with simple pulmonary metastasis disappeared without surgical resection after primary liver tumor resection and standard chemotherapy, the AFP levels returned to normal under monitoring, and the patients achieved clinical CR), 62 patients achieved PR, and the therapeutic efficacy was 81.0% (256/316). In 12 cases, there was PD, and 48 cases died (among these cases, two patients died of the second tumor leukemia and progression after treatment; one patient died of multiple hepatic recurrences with multiple bone, bone marrow, and intracranial metastasis after chemotherapy, radiotherapy, and APBSCT; one patient died of recurrence and progression after chemotherapy and liver transplantation due to multiple intrahepatic lesions; and one patient was complicated with giant metastasis of the right atrium and died due to cardiac arrest resulting from tumor thrombus falling off; all four children with small cell undifferentiated type). The Kaplan–Meier survival analysis showed that the OS was 95.3, 88.2, and 79.8% at 1 year, 3 years, and 5 years, respectively (Fig. 5), and the EFS was 91.1, 83.2, and 75.1%, respectively (Fig. 6). At the same time, we analyzed the survival rate of 82 older children whose age were over 3 years old (median age 7.33 years old) in the same period. Their 5-year OS and EFS were 71.4 and 60.5% respectively, both lower than those of Hb children under 3 years old. The 5-year OS among different subgroups according to the clinical factors was statistically analyzed, and the results with statistical differences are shown in Table 3.

**Table 3:** 5-year OS and single factor comparison of 316 children under 3 years of age with HB

	OS of five years (%)	χ^**2**^	P
Age (years-old)			
< 1	88.8	4.240	0.039
1–3	75.3		
AFP at first consultation (ng/mL)			
≤ 100	39.3	16.148	0.004
101–1000	90.3		
1001-1000,000	79.8		
> 1000,000	52.8		
Platelet count at first consultation (×10^9^/L)			
≤ 400	94.8	33.015	0.000
> 400	66.3		
Pathological typing			
Epithelial type	84.2	4.466	0.032
Mixed type	75.2		
The PRETEXT staging system			
Stage I	100	41.740	0.000
Stage II	96.5		
Stage III	77.9		
Stage IV	32.4		
Portal vein (P) invasion, Hepatic vein/vessel (V) invasion			
Y	61.0	20.340	0.000
N	85.4		
Distant metastasis (M)			
Y	69.1	7.355	0.007
N	94.6		
Multifocality (F)			
Y	56.1	7.041	0.021
N	89.7		

### Analysis of prognostic factors

According to the log-rank test, prognosis of HB was correlated with age, AFP level, platelet count, pathological typing, PRETEXT, portal vein/hepatic vein and vena cava invasion (P/V), distant metastasis (M), and multiple intrahepatic foci (F) (*p* < 0.05 in all) (Table 3). However, there was no statistical correlation between prognosis and sex, invasion of the extrahepatic adjacent tissues and organs (E), rupture of the tumor (R), and complete resection of the primary tumor (*p* > 0.05). The above clinical factors with statistical significance were introduced into the Cox regression model to perform multivariate analysis. This revealed that an AFP level of < 100 ng/mL (HR = 2.164, *p* = 0.020), a platelet count > 400 × 109/L (HR = 3.234, *p* = 0.006), PRETEXT IV (HR = 4.162, *p* = 0.001), vascular invasion (HR = 2.763, *p* = 0.012), and distant metastasis (HR = 2.112, *p* = 0.024) were the independent risk factors for the prognosis of HB in children, and the differences were statistically significant.

## Discussion

In recent years, international cooperation in the treatment of HB has made great progress through cooperative multicenter research [9]. However, distant metastasis occurs in most cases at the time of diagnosis of HB, which seriously reduces the post-therapeutic survival and prognosis in pediatric patients with HB. Therefore, it is essential to understand the relevant risk factors affecting the prognosis of HB in children, as this can guide the corresponding stratified treatment in clinical practice and achieve better clinical efficacy.

It has been reported that the 5-year OS of pediatric patients with HB can reach 75%, and the 5-year EFS has reached approximately 65% [10]. In the present study, children aged under 3 years (median age of 1.45 years old) with HB were studied with a median follow-up duration of 62 months. The results showed that the clinical therapeutic efficacy was 81.0%, and the 5-year OS and EFS were 79.8 and 75.1%, respectively, which were higher than the percentages reported internationally. In addition, this study also analyzed the survival rate of older HB children over 3 years old in the same period, and found that HB children under 3 years old had a better prognosis. Maibach et al. [11] believed that the older the diagnosis age of children with HB, the more obvious the adverse trend that affects their prognosis might be. The recent study confirmed this (*p* = 0.039). However, multivariate analysis indicated that age was not an independent risk factor for the prognosis of HB, which might be due to the fact that the present study focused on children aged under 3 years. This result can be verified in the future by expanding the age range of samples in the database. It is worth noting that in the present study, there were five cases of fetal onset of HB during pregnancy, and three cases were born with low birth weight. Maruyama et al. [12] investigated 15 children with low birth weight who were found to have fetal onset of HB during pregnancy and diagnosed with HB after birth, and suggested that children with low birth weight, especially those with very low birth weight, were prone to develop HB in the future, which might be another risk factor for HB.

In the present study, statistical analysis of the epidemiology of HB in China was conducted. Although there was no statistical difference in the survival of HB among different regions (*p* = 0.869), the analysis revealed that the incidence of HB in the coastal areas was higher than that in the inland areas. A reason for this might be that the industrial development in coastal areas was more advanced than that in the mainland, the air and water pollution were relatively serious, and the diet structure was mainly composed of seafood and bacon. In addition, low birth weight and parental tobacco using prior to or during pregnancy may increase risk of hepatoblastoma [13]. However, the results of the present study were limited to the statistical results of our single center. The incidence of HB might also be affected by many factors, such as medical resources and economic development in different regions. As our single center is located in North China, most of the patients came from the local region. It is expected that our center will cooperate with other centers in the future to conduct multi-center, large-scale epidemiological investigations.

As a tumor marker of HB, serum AFP not only has important prognostic significance in the initial diagnosis, but is also one of the important therapeutic indicators during the treatment [14]. In the present study, the prognosis of children with an AFP level < 100 ng/mL was worse than that of other groups (*p* < 0.05), which was an independent risk factor affecting the prognosis of HB. The results of Piotr Czaudernaa and Rebecka L Meyers et al. [15, 16] were consistent with those of the present study. In 2015, a study [17] showed that the increase in platelet count in the peripheral blood was correlated with infection, inflammatory disease, malignant tumor, and some chronic myeloid diseases. During the clinical practice, we found that some cases, especially those with stage IV, were prone to demonstrate an abnormal increase in platelet count at the initial diagnosis. The present study also confirmed that the increase in platelet count was another independent factor affecting the prognosis of HB.

It has been reported [18] that the prognosis of patients with HB of the mixed type is worse than that of patients with HB of the epithelial type, with the fetal type having the best prognosis, and that of the small-cell undifferentiated type being relatively poor. In the case analysis in the present study, it was found that different pathological types were correlated with the survival of pediatric patients. All four children whose pathologic type were small cell undifferentiated type eventually died with poor prognosis, which is consistent with the literature. However, the pathological type was not an independent risk factor for the prognosis. At present, SIOPEL believes that the PRETEXT in children with HB has an important prognostic value [11], and the advantage of the PRETEXT over postoperative staging such as the COG stage is that it is applicable to all children with HB, especially those without surgery, and it can predict the resectability of the tumor to a certain extent, while complete resection of the liver tumor is the key to the treatment of HB in children. The present study demonstrated that the OS of children with PRETEXT IV was significantly lower than that of pediatric patients with other stages. It also confirmed that the PRETEXT was significantly correlated with the prognosis.

Distant metastasis is prone to occur in HB and is an independent risk factor for the prognosis. The lung is the most common site of metastasis, most of which is from blood transmission. The tumor cells will stay to form metastasis as they reach the end of pulmonary vessels. Therefore, pulmonary metastasis usually occurs at the edge of the supply area at the end of the blood vessels of the bilateral lungs [19]. Of the 132 cases with distant metastasis in the present study, lung metastasis accounted for 80.3%. Moreover, single lung metastasis was more common than bilateral lung metastasis, and single marginal lung metastasis was more common than central lung metastasis, which is consistent with the literature.

## Conclusion

According to the results of multivariate analysis of the COX regression model, in addition to AFP level, platelet count, PRETEXT and distant metastasis, vascular invasion was an independent risk factor for the prognosis of HB in children. However, it should be noted that whether there was tumor rupture, infiltration of the extrahepatic adjacent tissues and organs, and multiple lesions in the liver, and whether the tumor can be completely resected had no clear effect on the prognosis (*p* > 0.05), which was not completely consistent with the relevant reports in the literature [20, 21]. It was speculated that the main reason for these differences might be that most of the children treated for HB in our center were in a late stage or refractory stages, which to some extent covers up the influence of these risk factors on the survival. Other studies have suggested that maternal hypertension during pregnancy, excessive amniotic fluid, smoking history, and birth weight < 1500 g might increase the incidence of HB [22]. Although these factors were not investigated in the present study, it is expected that clinical HB data of more centers will be collected in the future to establish a more scientific and perfect risk stratification system. Thus, the individualized and standardized treatment may be realized, and the survival and long-term prognosis of HB in children may be further improved, which will help in the rehabilitation of HB.

## Data Availability

We declared that materials described in the manuscript, including all relevant raw data, will be freely available to any scientist wishing to use them for non-commercial purposes, without breaching participant confidentiality.

## Abbreviations

HB: Hepatoblastoma
COG: Children’s Oncology Group
SIOPEL: International Childhood Liver Tumor Strategy Group
APBSCT: Autologous peripheral blood stem cell transplantation
EFS: Event-free survival
OS: Overall survival
AFP: Alpha-fetoprotein
CR: Complete remission
PR: Partial remission
PD: Progressed disease
P/V: Portal vein/hepatic vein and vena cava Invasion
M: Distant metastasis
F: Multiple intrahepatic foci
E: Invasion of the extrahepatic adjacent tissues and organs
R: Rupture of the tumor
